# Vitamin D deficiency and duration of COVID-19 symptoms in UK healthcare workers

**DOI:** 10.3389/fmed.2024.1494129

**Published:** 2024-11-25

**Authors:** Karan R. Chadda, Sophie A. Roberts, Sebastian T. Lugg, Aduragbemi A. Faniyi, Sian E. Faustini, Craig Webster, Joanne E. Duffy, Martin Hewison, Adrian Shields, Alex G. Richter, Dhruv Parekh, Aaron Scott, David R. Thickett

**Affiliations:** ^1^Acute Care Research Group, Department of Inflammation and Ageing, University of Birmingham, Birmingham, United Kingdom; ^2^Homerton College, University of Cambridge, Cambridge, United Kingdom; ^3^Clinical Immunology Service, Department of Immunology and Immunotherapy, University of Birmingham, Birmingham, United Kingdom; ^4^University Hospitals Birmingham National Health Service Foundation Trust, Birmingham, United Kingdom; ^5^Department of Metabolism and Systems Research, University of Birmingham, Birmingham, United Kingdom; ^6^National Institute for Health and Care Research (NIHR) Birmingham Biomedical Research Centre, Birmingham, United Kingdom

**Keywords:** vitamin D, viral symptoms, COVID-19, healthcare workers, long COVID

## Abstract

**Objectives:**

Vitamin D has a role in the innate immunity against pathogens and is also involved in mechanisms for reducing inflammation. VD deficiency (VDD) may increase COVID-19 infection susceptibility, however research is limited on the association between VDD and COVID-19 symptom prevalence and duration. The study aimed to determine whether VDD is a risk factor for the presence and extended duration of COVID-19 symptoms.

**Methods:**

Data was analyzed from NHS healthcare workers who isolated due to COVID-19 symptoms as a part of the COVID-19 convalescent immunity study between 12th to 22nd May 2020. Participants self-reported the presence and duration of viral symptoms. Anti-SARS-CoV-2 antibodies and vitamin D (25(OH)D_3_) serum levels were measured on day of recruitment. VDD was defined as 25(OH)D_3_ levels of < 30 nmol/l.

**Results:**

Of the 392 participants, 15.6% (*n* = 61) had VDD. VDD participants had more symptoms overall (*p* = 0.0030), including body aches (*p* = 0.0453), and extended duration of body aches (*p* = 0.0075) and fatigue (*p* = 0.0127). Binary logistic regression found that both VDD (OR 3.069, 95% CI 1.538–6.124; *p* = 0.001) and age (OR 1.026, 95% CI 1.003–1.049; *p* = 0.025) were independently associated with extended durations of body aches. VDD (OR 2.089, 95% CI 1.087–4.011; *p* = 0.027), age (OR 1.036, 95% CI 1.016–1.057; *p* < 0.001) and seroconversion (OR 1.917, 95% CI 1.203–3.056; *p* = 0.006), were independently associated with extended durations of fatigue.

**Conclusion:**

VDD is a significant independent risk factor for extended durations of body aches and fatigue in healthcare workers who isolated for COVID-19 viral symptoms. Vitamin D supplementation may reduce symptom duration and is thus an area for future research.

## Introduction

COVID-19, the respiratory RNA viral infection caused by Severe Acute Respiratory Syndrome coronavirus 2 (SARS-CoV-2), caused the most significant global health crisis since the 1918 influenza pandemic (1). The high transmissibility of this virus combined with vulnerable populations has led to over 7 million deaths globally (2). Common symptoms of COVID-19 include fever, cough, body aches, loss of taste and smell, sore throat, diarrhea, breathlessness, and fatigue (3). The severity and persistence of these COVID-19 symptoms have been found to impact health-related quality of life, suggesting a need for further research into approaches which alleviate such symptoms (4).

Vitamin D deficiency (VDD) causes dysregulation in both innate and adaptive immunity, thus increasing respiratory infection risks as is seen in influenza, tuberculosis, and common cold viruses (5, 6). For example, VDD upregulates pro-inflammatory cytokines like tumor necrosis factor alpha (TNFα), interleukin-6 (IL-6), and type 1 interferons (7). Vitamin D contributes to viral infection protection by decreasing these inflammatory cytokines (7), increasing antimicrobial peptide (AMP) production in the respiratory epithelium (8), and decreasing pulmonary vasoconstriction (7).

A meta-analysis showed low levels of vitamin D have been linked to an increased risk of COVID-19 infection and increased disease severity, although the included studies were observational and heterogenous (9). VDD has been associated with a longer disease course, more severe lung involvement and higher risk of death in elderly patients with COVID-19 (10). Whilst research has shown links between vitamin D supplementation and COVID-19 hospitalization and severity (11), there is a notable lack of research available regarding the impact of VDD on the duration of COVID-19 symptoms. Analysis of Neale's D-Health trial demonstrated that while monthly bolus doses of 60,000 IU of vitamin D did not reduce risk of acute respiratory tract infections, it unsignificantly decreased symptom duration (12).

There is limited research available on the impact that patient vitamin D status has on the duration of COVID-19 symptoms. We hypothesized that VDD is a significant independent risk factor for extended durations of COVID-19 symptoms. The aim of this study was to assess the relationship between VDD and COVID-19 symptom duration, as well as the relationship between VDD and COVID-19 symptom quantity.

## Methods

As a part of the observational COVID-19 convalescent immunity (COCO) study, healthcare workers were recruited between 12th to 22nd May 2020 from the University Hospitals Birmingham NHS Foundation Trust (UHBFT). Consent has been obtained from each patient after full explanation of the purpose and nature of all procedures used. All participants were isolating due to symptoms suggestive of COVID-19 during the first wave of the pandemic, and therefore pre-dated mass testing (PCR or lateral flow) outside hospitalized patients. This study was approved by the London—Camden & Kings Cross Research Ethics Committee (20/HRA/1817).

Blood samples were collected from all participants for anti-SARS-CoV-2 spike glycoprotein antibody analysis and to determine serum vitamin D status. The median interval between symptom onset and sample collection was 48 days (13). Anti-SARS-CoV-2 spike glycoprotein antibody used a combined IgG, IgA, IgM ELISA (The Binding Site, Product code: MK654); this assay has 98.6% sensitivity and 98.3% specificity (14). COVID-19 seroconversion was used to signify SARS-CoV-2 infection prior to the study.

Serum 25(OH)D3 was quantified by mass spectrometry. Participant vitamin D levels were categorized as VDD when serum vitamin D concentration < 30 nmol/L, as per the UK National Osteoporosis Society guidance (15). Alongside 25(OH)D3 levels, demographic details were recorded including age, gender, BMI, and ethnicity. The presence or absence of several symptoms was self-reported by each participant alongside the start and end date. These eight symptoms consisted of cough, breathlessness, fever, sore throat, body aches and pains, fatigue, lack of sense of smell, and diarrhea.

Data was analyzed using IBM SPSS Statistics (version 27) and GraphPad Prism. Continuous data in each study were tested for normality using the Shapiro-Wilk test. Mann-Whitney *U*-test was used to assess significant differences in non-parametric continuous data between groups, while Fisher's exact test was used to detect significant differences in categorical data between groups. Binary logistic regression analysis was used to determine significant independent risk factors within a multivariate model, with receiver operator characteristic (ROC) analysis used to determine the effectiveness of the model. *P*-value < 0.05 denoted a significant result and subsequent rejection of the null hypothesis.

## Results

**Figure 1:**
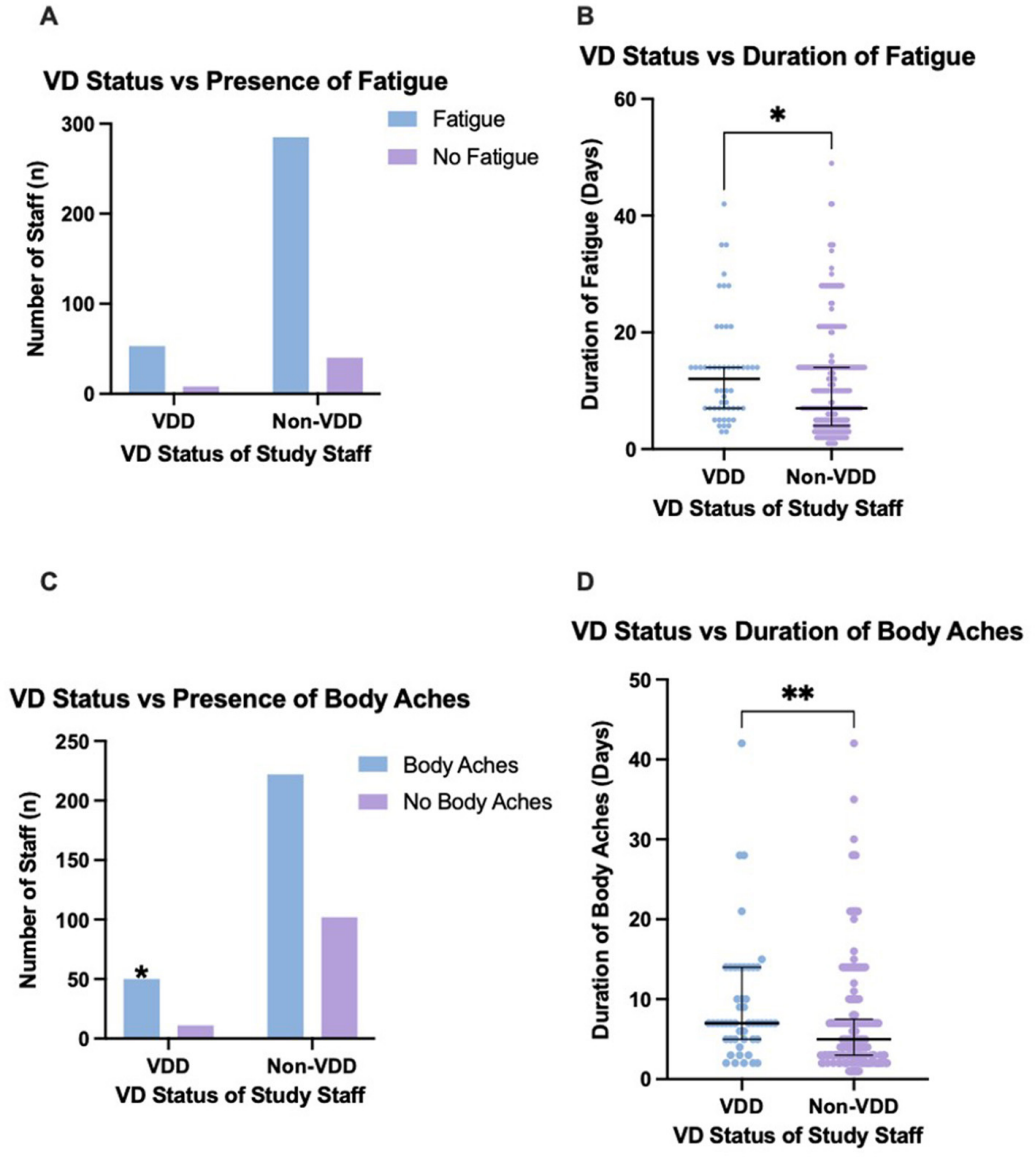
The presence of body aches and fatigue between vitamin D deficient and non-vitamin D deficient staff, and the duration of these symptoms. **(A)** No significant difference in presence of fatigue between VDD and non-VDD staff. **(B)** VDD staff experienced a significantly greater duration of fatigue (*p* = 0.0127). **(C)** VDD staff had a significantly higher presence of body aches. **(D)** VDD staff experienced a significantly greater duration of body aches (*p* = 0.0075). Error bars display the median and IQR. Pairwise comparisons with asterisks indicates the significant differences (*p ≤ 0.05; **p ≤ 0.01). Graphs produced in GraphPad Prism. VD, vitamin D.

The baseline demographic details of this cohort have been previously published (13). Of the 392 healthcare workers studied, 61 (15.6%) had VDD. There were 8 symptoms reported by the symptomatic healthcare staff including cough (*n* = 268, 68.4%), breathlessness (*n* = 185, 47.2%), fever (*n* = 234, 59.7%), sore throat (*n* = 196, 50%), body aches and pains (*n* = 272, 69.4%), fatigue (*n* = 338, 86.2%), lack of sense of smell (*n* = 161, 41.1%) and diarrhea (*n* = 114, 29.1%). Healthcare workers with VDD had a higher prevalence of body aches and pains than those without VDD (82.0 vs. 67.1%; *p* = 0.0453) (Table 1; Figure 1). There were no significant differences in the other seven symptoms amid VDD and non-VDD staff. Although self-reported fever showed higher prevalence in VDD participants, this did not reach significance (*p* = 0.0626).

**Table 1:** Symptom present and absence in vitamin D deficient and non-vitamin D deficient staff.

		**Total**	**Vitamin D deficient**	**Non-vitamin D deficient**	***p*-value**
Symptomatic staff (*n*)		392	61	331	
Cough (% of staff *n*)	Yes	268 (68.4%)	46 (75.4%)	223 (67.4%)	0.44
	No	115 (29.3%)	15 (24.6%)	100 (30.2%)	
Breathlessness (% of staff *n*)	Yes	185 (47.2%)	28 (45.9%)	157 (47.4%)	0.78
	No	201 (51.3%)	33 (54.1%)	168 (50.8%)	
Fever (% of staff *n*)	Yes	234 (59.7%)	44 (72.1%)	190 (57.4%)	0.06
	No	149 (38.0%)	17 (27.9%)	132 (39.9%)	
Sore throat (% of staff *n*)	Yes	196 (50.0%)	37 (60.7%)	159 (48.0%)	0.12
	No	189 (48.2%)	24 (39.3%)	165 (49.8%)	
Diarrhea (% of staff *n*)	Yes	114 (29.1%)	23 (37.7%)	91 (27.5%)	0.17
	No	271 (69.1%)	38 (62.3%)	233 (70.4%)	
Fatigue (% of staff *n*)	Yes	338 (86.2%)	53 (86.9%)	285 (86.1%)	0.83
	No	48 (12.2%)	8 (13.1%)	40 (12.1%)	
Body aches/pains (% of staff *n*)	Yes	272 (69.4%)	50 (82.0%)	222 (67.1%)	0.0453
	No	113 (28.8%)	11 (18.0%)	102 (30.8%)	
Lack of sense of smell (% of staff *n*)	Yes	161 (41.1%)	31 (50.8%)	130 (39.3%)	0.15
	No	215 (54.8%)	29 (47.5%)	186 (56.2%)	

390 out of the 392 staff were analyzed. In cases where total patients experiencing symptom and no symptom does not add up to 390, this is due to instances in the datasheet where the symptom is “not stated”, “unknown”, or the result was inputted in a way in which either outcome could not be assumed e.g., “yes no”. Fever was defined as a temperature >37.8°.

Those with VDD were significantly more likely to experience a greater median total number of symptoms when compared to non-VDD staff (median 5, IQR 4–7 vs. median 4, IQR 3–6; *p* = 0.0030) (Table 2). VDD staff also had a statistically significantly higher duration of both body aches (median 7 days, IQR 5–14 vs. median 5 days, IQR 3–7.5; *p* = 0.0075) and fatigue (median 12 days, IQR 7–14 vs, median 7 days, IQR 4–14; *p* = 0.0127) (Table 3; Figure 1). VDD did not influence the duration of the other six symptoms analyzed, such as cough and fever.

**Table 2:** Number of symptoms experienced in vitamin D deficient and non-vitamin D deficient staff.

		**Total**	**Vitamin D deficient**	**Non-vitamin D deficient**	**p-value**
Symptomatic staff *n*		392	61	331	
Number of 8 symptoms experienced (% of staff *n*)	0–4 symptoms	192 (49%)	21 (34.4%)	171 (51.7%)	0.01
	5–8 symptoms	198 (50.5%)	40 (65.6%)	158 (47.7%)	
Number of eight symptoms experienced, median (IQR)		5 (3–6)	5 (4–7)	4 (3–6)	0.003

390 out of the 392 symptomatic staff were included in the analysis as two staff did not have symptom data available. IQR, interquartile range.

**Table 3:** Duration of symptoms and vitamin D status.

**Symptom duration**	**Total**	**Vitamin D deficient**	**Non-vitamin D deficient**	**p-value**
All symptoms, median (IQR) days	13 (7–21)	14 (7.5–22)	13 (7–21)	0.60
Cough, median (IQR) days	10 (5–20.5)	7 (5–15.5)	10 (4–21)	0.86
Breathlessness, median (IQR) days	7 (3–14)	7 (4–14.25)	7 (3–14)	0.28
Fever, median (IQR) days	3 (2–7)	5 (2–7)	5 (2–7)	0.26
Sore throat, median (IQR) days	5 (3–7)	5.5 (3–7)	5 (3–7)	0.77
Diarrhea, median (IQR) days	3 (2–4.5)	2 (2–5)	3 (2–4)	0.90
Fatigue, median (IQR) days	8 (5–14)	12 (7–14)	7 (4–14)	0.01
Body aches, median (IQR) days	6 (3–10)	7 (5–14)	5 (3–7.5)	0.0075
Lack of sense of smell, median (IQR) days	14 (7–21)	14 (7–21)	14 (7–21)	0.96

A summary of the analysis on the duration of each symptom in the 390 symptomatic hospital workers, compared to their VD status. IQR, interquartile range.

Backwards logistic regression models were generated to determine the factors significantly associated with an extended duration of fatigue and body aches. These models determined the effects of VDD, age, BMI, gender, ethnicity, and seroconversion on the likelihood that individuals with suggestive COVID-19 experience body aches and fatigue for a longer duration. For each of these symptoms, the median duration was chosen to dichotomise the staff into two groups, in order to perform the binary logistic regression.

Both VDD (OR 3.069, 95% CI 1.538–6.124; *p* = 0.001) and age (OR 1.026, 95% CI 1.003–1.049; *p* = 0.025) were significant independent risk factors for experiencing a longer duration of body aches (Table 4). VDD staff were over 3 times more likely to experience a longer duration of body aches than non-VDD staff. The goodness-of-fit test of this model remained non-significant during these steps, with a *p*-value close to 1 showing a good fit for the final model (Hosmer and Lemeshow *p* = 0.981). The overall predictive power of this model was 62.8% (95% CI 56–69.6, SE 3.5%; *p* < 0.001) as indicated by the area under the ROC curve.

**Table 4:** Significant independent variables associated with longer body ache duration.

**Variable**	**Estimate**	**SE**	***p*-value**	**OR**	**95% CI**
Vitamin D deficiency	1.121	0.352	0.001	3.069	1.538–6.124
Age (years)	0.025	0.011	0.03	1.026	1.003–1.049
Constant	−1.209	6.007	0.01		

The median duration of body aches was 6 days, and this was chosen as the cut off for categorizing durations in the binary logistic regression. SE, standard error; OR odds ratio; CI, confidence interval.

VDD (OR 2.089, 95% CI 1.087–4.011; *p* = 0.027), age (OR 1.036, 95% CI 1.016–1.057; *p* = < 0.001) and seroconversion (OR 1.917, 95% CI 1.203–3.056; *p* = 0.006), were significant independent risk factors for experiencing a longer duration of fatigue (Table 5). VDD staff were over 2 times more likely to experience a longer duration of fatigue than non-VDD staff. Age is a significant independent risk factor for a longer duration of both symptoms, suggesting VDD is not solely responsible. For every 1-year increase in age, the odds are 2.6% higher (OR = 1.026) for facing a longer duration of body aches and 3.6% higher (OR = 1.036) for facing a longer duration of fatigue. The goodness-of-fit test of this model remained non-significant during these steps, with a *p*-value close to 1 showing a good fit for the final model (Hosmer and Lemeshow *p* = 0.717). The overall predictive power of this model was 66.4% (95% CI 60.4–72.4, SE 3.1%; *p* < 0.001) as indicated by the area under the ROC curve.

**Table 5:** Significant independent variables associated with longer fatigue duration.

**Variable**	**Estimate**	**SE**	***p*-value**	**OR**	**95% CI**
Vitamin D deficiency	0.736	0.333	0.03	2.089	1.087–4.011
Age (years)	0.035	0.010	< 0.001	1.036	1.016–1.057
Seroconversion	0.651	0.238	0.006	1.917	1.203–3.056
Constant	−1.908	0.464	< 0.001		

The median duration of fatigue was 8 days, and this was chosen as the cut off for categorizing durations in the binary logistic regression. SE, standard error; OR odds ratio; CI, confidence interval.

## Discussion

This study has shown that vitamin D deficiency (VDD) is associated with a greater total quantity of COVID-19 symptoms for lengthier durations. Our analysis revealed VDD as a significant risk factor for body aches as a symptom in people isolating for suggestive COVID-19. VDD is also a significant independent risk factor for experiencing a longer duration of both body aches and fatigue.

The binary logistic regression revealed that age is also a significant independent risk factor for longer duration of both symptoms, suggesting VD status is not solely responsible. This is supported by another study showing that older age is associated with a slower resolution of COVID-19 symptoms (16). This may be due to the gradual decline of the immune system that accompanies aging, also known as immunosenescence (17). This hinders detection of pathogens and subsequent alert signaling and clearance (17), which may mean it takes longer to clear the SARS-CoV-2 infection. However, in both models, the odds ratio (OR) for VDD is much further from 1 than the OR for age, which is only very slightly > 1. This indicates it is more likely that the relationship between VDD and extended duration is causal than the relationship between age and extended duration (18). The regression analysis also revealed seroconversion to be a significant independent risk factor for extended fatigue duration. However, the OR for VDD (2.089) is further from 1 than seroconversion (1.917), suggesting it is more likely that the relationship between VDD and longer fatigue duration is causal, compared to the relationship between seroconversion and duration (18).

VDD staff were over 3 times more likely to experience a longer duration of body aches than non-VDD staff. The anti-inflammatory properties of vitamin D can become dysregulated with deficiency, possibly accounting for this extended duration of body aches. Vitamin D has been shown to decrease the production and release of pro-inflammatory cytokines by preventing translocation of transcription factor nuclear factor kappa B (NFκB) to the nucleus (19). Vitamin D also inhibits pro-inflammatory T helper type 1 (Th1) cell development, thereby inhibiting the production of interleukin-2 (IL-2) and interferon-γ (INFγ) (20). By suppressing pro-inflammatory Th1 cells, vitamin D promotes the transition of these cells into the suppressive Th2 phenotype which produces IL-10, an anti-inflammatory cytokine (21). Vitamin D also suppresses Th17 cells, which are responsible for producing the inflammatory cytokine IL-17 (20).

There is evidence to suggest that body aches, specifically myalgia in colds and flu, are caused by prostaglandin E2 (PGE2) production in reaction to circulating cytokines (22); this is likely also the case in COVID-19. Vitamin D inhibits COX-2, which catalyzes synthesis of prostaglandins (23). Vitamin D also stimulates expression of 15-prostaglandin dehydrogenase (15-PGDH), which catalyzes the degradation of prostaglandins (23). Low vitamin D levels are inversely correlated with high IL-6 levels in COVID-19 patients (24). This increase in cytokines, along with less COX-2 inhibition and less 15-PGDH expression, may result in higher prostaglandin levels in VDD individuals with COVID-19. This is merely a speculative explanation for the increased duration of time taken for VDD staff to recover from symptomatic body aches. It is important to note that as prostaglandins are short-lived molecules, the extended myalgia is most likely due to multiple inflammatory mediators which are dysregulated in VDD.

VDD staff were also over two times more likely to experience a longer duration of fatigue than non-VDD staff. Fatigue is the most typical symptom linked with an activated immune system (25) and is also associated with VDD (26). It is therefore not surprising that VDD, coupled with an activated immune system after viral infection, is associated with longer fatigue duration than in those who are non-VDD. A confounding factor here is the self-reported recording of fatigue. Given that the study was undertaken at the start of the pandemic, it is not unexpected that overworked healthcare workers were reporting fatigue, as they were likely working long hours inside hospitals. Perhaps if the study were to be repeated in individuals outside the healthcare industry now that the pandemic has passed its peak, the results may be different. Although the fatigue could be due to this increased workload, the multiple regression shows that COVID-19 seropositive staff were almost twice as likely (OR = 1.917) to experience a longer duration of fatigue. This suggests that COVID-19 infection is in fact significant in causing longer symptomatology, alongside VDD. However, a limitation is that we did not have non-COVID virology data to compare this to.

Whilst previous research has shown links between vitamin D supplementation and COVID-19 hospitalization and severity (11), there is a notable lack of research available regarding VDD's impact on COVID-19 symptom duration. Analysis of Neale's D-Health trial demonstrated that while monthly vitamin D supplementation did not reduce risk of acute respiratory tract infections, it marginally decreased symptom duration (12). However, this was not significant enough to be clinically relevant.

In a RCT on patients with moderate to severe COVID-19 randomized to receive a single dose of vitamin D3 (200,000 IU) or placebo, there was no significant difference between groups in hospital length of stay, mortality, admission to ICU or need for mechanical ventilation (27). However, it is important to note that the trial was underpowered for its primary outcome of hospital length of stay (28). Future studies should determine whether preventative daily vitamin D supplementation in VDD individuals before infection could be more effective than a singular high-dose bolus in reducing symptom duration and hospital stay in severe COVID-19 patients.

The COVIT-TRIAL was a multicentred RCT testing high dose vs. standard dose vitamin D on mortality rates of COVID-19 patients and showed a significantly higher 14-day survival in the high dose group but no difference in mortality at 28 days (29). Another RCT investigated whether extended-release calcifediol (ERC) affected symptom resolution time in patients with mild to moderate COVID-19. In the full analysis, symptom resolution time was unchanged between groups. In the per-protocol analysis, respiratory symptoms resolved 4 days earlier when serum vitamin D was elevated above baseline (30). A meta-analysis on nine RCTs showed that vitamin D treatment was associated with a reduced risk of ICU admission but no significant changes in other hospital or laboratory outcomes (31). A retrospective analysis showed that micronutrient supplementation, including with vitamin D, was not associated with a lower risk of experiencing COVID-19 symptoms in young adults, although the study was limited by a small sample size and recall bias (32).

Long COVID is characterized by multiple persisting symptoms for more than 12 weeks after COVID-19 infection and it has become a significant health problem (33, 34). Symptoms can be widespread and include cardiorespiratory, gastrointestinal, musculoskeletal and neurological systems, causing an overall decline in quality of life (35). Given its immunomodulatory and anti-inflammatory properties, it a hypothesized that vitamin D modulation may be a useful therapeutic avenue for long COVID (36, 37). Patients with long COVID have been shown to have lower vitamin D levels at 6 months compared to COVID-19 survivors without long COVID, and the same study showed vitamin D levels were an independent risk factor for long COVID (38). Correcting vitamin D levels may therefore warrant further investigation for long COVID.

## Limitations

This study has several limitations. As the staff participant data was collected in May 2020, different results may have been obtained if data was collected at a different time of year; vitamin D levels are typically lower in autumn and winter. A previous study showed that 86% of adults had normal vitamin D at some point during the year but only 49% maintained normal levels in both autumn and spring, indicating its seasonal variability (39). Furthermore, the study was conducted during the first wave of the pandemic, including the early variant (Alpha) of COVID-19. Newer variants have since emerged with alternative symptom presentation, such as nausea, which were not investigated in this study. It has been shown that certain variants, such as Delta lineages AY.126 and AY.43, and Omicron sublineages BA.1.17, BA.2.56, and BA.5.1, are associated with more severe persistent symptoms (40).

The data was also collected before COVID-19 vaccines had become available. With vaccines shown to reduce the severity, presence, and duration of COVID-19 symptoms (41), vitamin D levels may now have less of an effect on fatigue and body ache duration than this analysis suggests. There are conflicting reports on the efficacy of COVID-19 vaccination in reducing the incidence of long COVID (42). For example, one study showed no difference in long COVID incidence between vaccinated and unvaccinated people (43). However, other studies have shown a reduction in long COVID risk with vaccination (44, 45). Low vitamin D levels have been associated with an impaired long-term response to COVID-19 vaccination (46). Furthermore, vitamin D supplementation has been shown to reduce post-vaccination side effects and boost the immunoglobulin G response to COVID-19 (47). Lastly, the hospital staff all had mild COVID-19; the study does not give insight into the role of VDD in severe COVID-19 symptom duration. An observational study suggested a higher risk of severe COVID-19 is associated with low vitamin D (48). Interestingly, another study did not find an association between severe long COVID and low levels of vitamin D (49). Future research therefore needs to consider both mild and severe cases.

## Conclusion

Vitamin D deficiency (VDD) is an extremely common condition worldwide, affecting approximately one billion people. In healthcare workers who had isolated due to COVID-19 symptoms, VDD was a significant independent risk factor for an extended duration of body aches and fatigue. Vitamin D supplementation may be an extremely cost-effective and viable approach in reducing this risk and managing some symptoms of long COVID, and this should be an area of future research.

## Data Availability

The raw data supporting the conclusions of this article will be made available by the authors, without undue reservation.
